# Biodegradable chitosan/PVA-based hydrogel incorporating green synthesized silver nanoparticles for wound healing applications

**DOI:** 10.1186/s13065-025-01564-5

**Published:** 2025-07-03

**Authors:** Abdelrhman Ragab, Nourhan El-Badry, Nouran Tamer, Ahmed Naas, Ahmed Hamdy, Samar H. Tawakey, Abdelrahim H. A. Hassan, Alyaa I. Salim

**Affiliations:** 1 School of Biotechnology, Nile University, Sheikh Zayed City, Giza 12588 Egypt; 2 Nanoelectronics Integrated Systems Center (NISC), 26th of July Corridor, Nile University, Sheikh Zayed City, Giza 12588 Egypt; 3 City University Ajman, Ajman, United Arab Emirates

**Keywords:** Antibacterial activity, Biocompatible, Chronic wound, Hydrogel film, Silver nanoparticles

## Abstract

Chronic wounds pose significant healthcare challenges globally. The need for more effective strategies for wound healing applications has led to the exploration of numerous emerging technologies. The current study investigates the wound-healing potential of green synthesized silver nanoparticles (AgNPs) using *Aloe Vera* and green tea loaded on polyvinyl alcohol (PVA)/chitosan hydrogel. To this end, different hydrogel films incorporating various types of AgNPs were prepared. XRD, SEM, and EDX confirmed good integration of the crystallographic structure of the green synthesized nanomaterial and indicated smooth surface morphology of the films. The results of the biodegradability test showed that the *Aloe Vera* synthesized AgNPs hydrogel patch exhibited a high degradation rate with (22%) weight loss after 30 days. The results of in vitro antimicrobial testing and cytotoxicity assays revealed that *Aloe Vera*-synthesized AgNPs possess higher antibacterial activity against *Escherichia coli* and *Staphylococcus aureus* with high cell viability (82%). The in vitro release of AgNPs showed a gradual release of AgNPs with stabilization. The water vapor transmission rate was found to range between 1388.89 g/m2/day and a moisture content of 7.73%. The tensile stress and elongation at break were found to range between 69.14-67-13 MPa and 4.84%-4.34%, respectively, indicating significant mechanical properties of the films. Overall, the results proved that green synthesized AgNPs hydrogel patches, especially using *Aloe Vera*, provide the optimal properties for wound healing, combining good moisture utilization, stability, and holding potential as a biodegradable, antibacterial wound dressing.

## Graphical Abstract

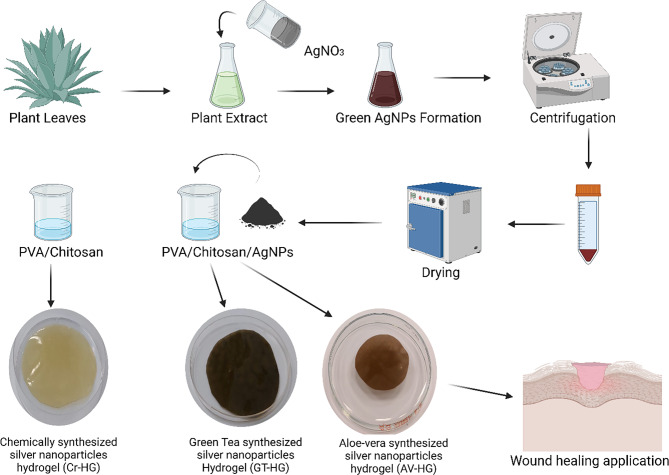

## Introduction

Many individuals experience various forms of skin epidermal damage each year, including burns, ulcers, and other traumatic events that result in the formation of acute or chronic wounds [1]. Chronic wounds, which fail to heal within an expected period, pose a significant challenge to healthcare systems worldwide, affecting an estimated 2.5% of the population. These wounds, often associated with diabetes, vascular disease, and aging, result in infection and increased morbidity and mortality [2]. Due to these factors, this widespread issue poses enormous economic and social challenges to global healthcare systems, emphasizing the critical need for innovative and effective wound-healing solutions [3].

**Fig. 1:**
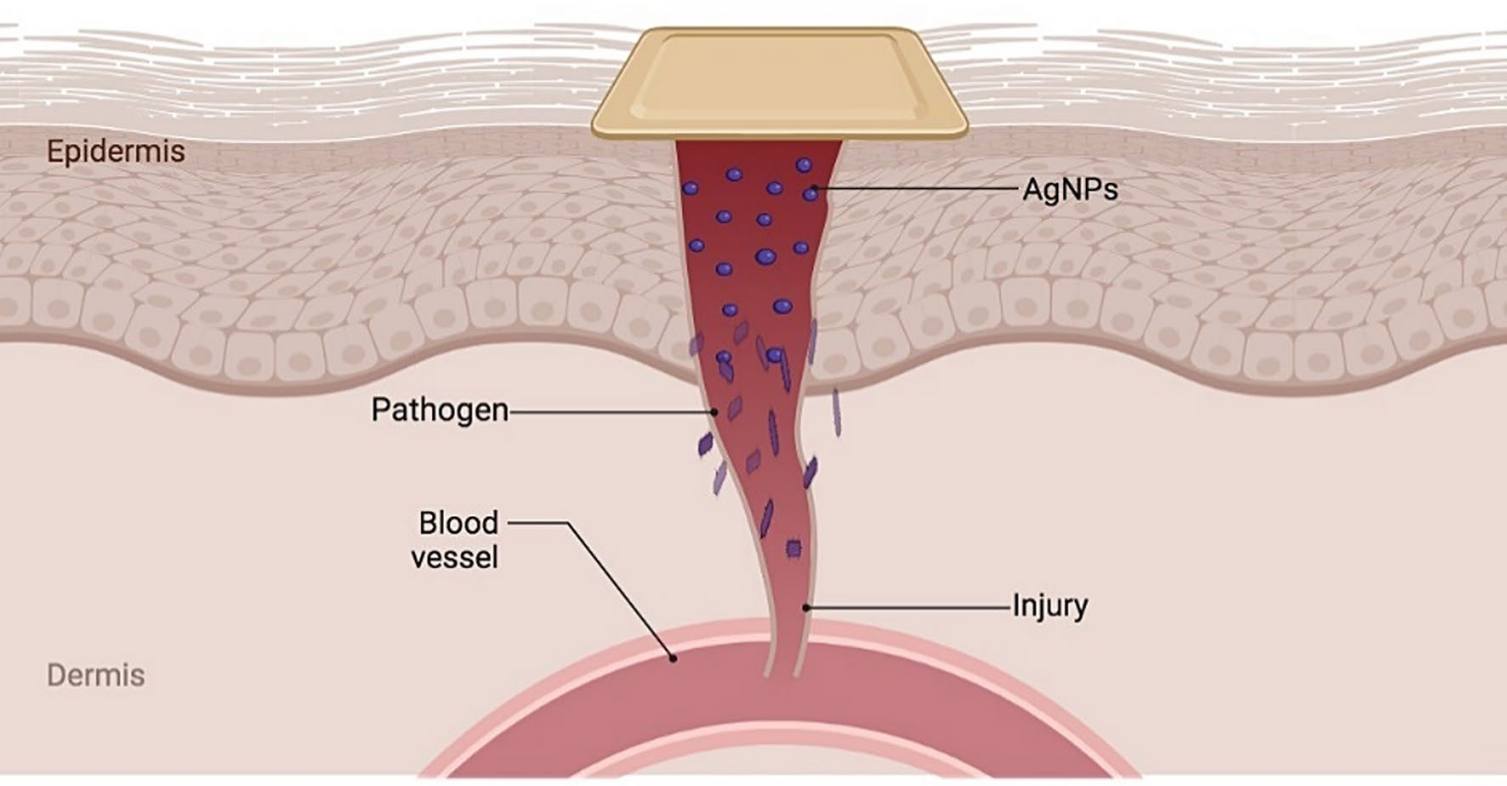
Mechanism of action of silver nanoparticles (AgNPs) and the activity of chitosan to promote wound closure by preventing bacterial colonization, and inflammation and stimulating the cells at the wound site to accelerate the healing

Silver nanoparticles (AgNPs) have emerged as a promising material for wound healing due to their potent antimicrobial properties and ability to release silver ions in a controlled manner. These ions can inhibit bacterial growth, reduce inflammation, promote tissue regeneration, and enhance cell proliferation and migration, as shown in Fig. 1. That makes AgNPs a valuable component in wound care applications [4]. The synthesis of AgNPs can be achieved through various methods, including chemical, physical, and biological approaches. Chemical synthesis often involves the use of toxic chemicals, which can pose environmental and health risks. Physical methods, while effective, can be energy-intensive and costly. In contrast, the green synthesis of AgNPs offers several advantages over these conventional methods. This eco-friendly approach utilizes non-toxic, renewable reagents, such as plant extracts, to synthesize nanoparticles [5]. *Aloe Vera* (*Aloe barbadensis miller*) and green tea (*Camellia sinensis*) are particularly effective biological agents of significant values in the green synthesis of nanoparticles. *Aloe Vera*, known for its anti-inflammatory and wound-healing properties, contains bioactive compounds that can reduce the cytotoxicity of AgNPs [6]. Similarly, green tea is rich in polyphenols and antioxidants, can enhance the antimicrobial efficacy of AgNPs by penetrating bacterial cell walls and subsequently change the structure of the cell membrane, while promoting biocompatibility [7]. These advantages make green-synthesized AgNPs highly suitable for wound healing applications.

Hydrogels are ideal for wound dressings because they can incorporate a wide range of bioactive agents and can be treated under mild conditions also for wounds ranging from dry to lightly exuding and can be used to degrade slimes on the wound surface Hydrogels can be made from many natural polymers including proteins such as collagen, gelatin, and polysaccharides such as starch, alginate, and agarose [8]. Hydrogels, composed of materials like chitosan (CS) and polyvinyl alcohol (PVA), are ideal carriers for AgNPs in wound healing applications. CS is derived from marine sources and is used on a commercial scale in the design and formulation of dosage forms. It is semi-synthetic and made from chitin. It has piqued the interest of researchers around the world due to its biodegradability and ability to be molded into films, blends, coatings, composites, and nanotechnology-enabled profiles. Also, CS could accelerate the wound-healing process by stimulating inflammatory cells, macrophages, and fibroblasts [3]. PVA is a vinyl polymer with carbon-carbon linkages. It is water soluble and biodegradable. It also has high biocompatibility and can self-crosslink due to the hydroxyl groups on the side chains. Together, CS and PVA materials form a hydrogel matrix that can effectively deliver green-synthesized AgNPs to the wound site as shown in Fig. 1, promoting healing through sustained antimicrobial activity and moisture retention [1].

The current study aimed to investigate the wound-healing potential of green synthesized AgNPs using *Aloe Vera* and green tea loaded on a CS/PVA hydrogel matrix to develop a biocompatible and biodegradable wound-healing patch. A range of tests was conducted to assess the suitability of films for potential use in wound healing, including measurements of thickness, weight fluctuation, folding endurance, moisture content, moisture uptake, swelling ratio, water vapor transmission rate (WVTR), tensile strength, and elongation to break. Characterization experiments using SEM, EDX, XRD, and DSC were also carried out. Additionally, hydrogel film formulations were subjected to stability testing, antibacterial activity testing, and in-vitro drug release. The idea of creating physically crosslinked hydrogels of PVA and CS without the use of any hazardous organic chemicals or solvents is what makes this research novel. This innovative approach leverages the synergistic properties of green-synthesized nanoparticles and hydrogels to create an effective and eco-friendly wound care solution.

## Experimental section

### Materials

Chitosan (Low molecular weight, 75.0% Deacetylated) was purchased from Alpha Chemika, Mumbai, India. PVA (molecular weight, approx. 115,000 Da, 98.9-mole percent hydrolyzed) was purchased from Loba Chemicals Pvt. Ltd. Mumbai, India. Glacial acetic acid (extra pure) was purchased from Amichem Research Lab, Dehradun, India. Silver nitrate (AgNO_3_), NaCl, and KCl were purchased from ACG, Cairo, Egypt. Green tea and *Aloe Vera* were procured from a local market in Cairo, Egypt. Calcium chloride (CaCl_2_) and sodium hydroxide (NaOH) were purchased from SRL, India.

Test strains: *Escherichia coli* and *Staphylococcus aureus* strains were obtained from the culture collection at Microbiology laboratory, School of Biotechnology, Nile University, Egypt.

### Green synthesis of silver nanoparticles from green tea

The green synthesis of AgNPs from green tea (GT) was prepared according to previous study [7]. For GT extraction, 600 mL of distilled water was added to 20 g of dried, ground GT leaves. The mixture was heated for 30 min at 50 °C under magnetic stirring. Subsequently, the mixture was cooled and filtered, and the supernatant was used for the synthesis of nanoparticles. For GT-based AgNPs synthesis, 125 mL silver nitrate solution (0.01 M) was added to 20% aqueous GT extract solution dropwise. During this process, the solution was mixed vigorously. Then, the solution was heated for 30 min at 50 °C to increase AgNPs yield, and 21 mL NaOH was added to the mixture till the formation of GT AgNPs denoted by the change of yellowish green tea into a brown color. GT AgNPs were separated by centrifugation at 6000 rpm, washed with water until it became clear then with 70% ethanol once, and allowed to dry at 40**°**C. A small portion of the suspension obtained was used for UV-Visible spectroscopic analysis.

**Green synthesis of silver nanoparticles from*Aloe Vera***.

The green synthesis of AgNPs from *Aloe Vera* has been carried out based on the previous report with some modifications [6]. An *Aloe Vera* extract solution was prepared by washing the leaves of *Aloe Vera* with distilled water, followed by 30 gm of finely chopping the leaves and boiling them in 300 mL distilled water for 20 min. Then, the mixture cooled and filtered. To synthesize *Aloe Vera*-based AgNPs, 0.3 mol of AgNO_3_ was dissolved in 20 mL of distilled water. AgNO_3_ solution was then mixed with 20 mL *Aloe Vera* extract solution with constant stirring at room temperature (25 ^0^C) for 30 min, the grey color formation indicates the synthesis of AgNPs. The centrifugation of *Aloe Vera*-based AgNPs was done at 6000 rpm, washed with water till it became clear then with 70% ethanol once, and allowed to dry at 40**°**C. A small portion of the obtained suspension was used for UV-Visible spectroscopic analysis.

From the literature survey *Aloe Vera* has long been recognized as a medical plant that combats infection, reduces inflammation, and heals burns and wounds. The inclusion of polysaccharides, amino acids, proteins, enzymes, terpenoids, flavonoids, vitamins, phenols, and other metabolites in its gel and plant extracts enhances its potential to reduce silver ions and form antimicrobial AgNPs [9]. The mechanism of action is based on chemicals found in *Aloe Vera* extracts that cause steric repulsion between silver nuclei by reducing the overall surface energy, preventing nanoparticles from aggregating. This biological process is faster than other chemical methods, and the basic components are easily obtained and inexpensive [10, 11]. 

Moreover, UV-Vis spectra and XRD measurements provide proof that silver ions are reduced into nanoparticles [12]. 

### Chemical synthesis of silver nanoparticles

In 2016,The synthesis of AgNPs using chemical method was performed according to Gudikandula and Maringanti [13]. All reagents were dissolved in double distilled water. A volume of 50 mL of a 1 × 10^− 3^ M AgNO_3_ solution was brought to a boil. Then, 5 mL of a 1% trisodium citrate solution was added dropwise to the boiling mixture with vigorous stirring. The heating process persisted until the color changed to pale brown. The solution was then removed from the heat and stirred until it reached room temperature, precipitated, and allowed to dry at 40 °C. A small portion of the obtained suspension was used for UV-Visible spectroscopic analysis. In contrast, our study not only involves the green synthesis of AgNPs from green tea or *Aloe Vera* but also includes a comparative study with chemically synthesized AgNPs. This allowed to investigate how different biological reducing agents affect the morphological and antimicrobial properties of the resulting nanoparticles.

### Characterization of AgNPs using UV-Vis spectroscopy and XRD

The optical properties of AgNPs were determined using a double-beam UV-Vis spectrophotometer (Peak, 425) with absorbance around 420 nm [14]. Further characterization of AgNPs was carried out by XRD.

### Preparation of hydrogel films incorporating AgNPs

Hydrogel films were prepared according to the method of Chopra et al. (2022), with modifications [1]. Four different hydrogel films were prepared. For the control hydrogel (Cr-HG), 0.8 g of CS was dissolved in a solution of 3% v/v of acetic acid while being constantly stirred for two hours. Meanwhile, 0.5 g of PVA was dissolved in 10 mL of distilled water with persistent stirring for four hours at 50 °C. Then CS and PVA solutions were mixed using a magnetic stirrer, and the resulting hydrogel mixture was poured into petri plates and left for 2 days at room temperature (25 ^0^C) to achieve the desired patch thickness and properties. For hydrogel incorporating chemically synthesized AgNPs (Ch-HG), chemically synthesized AgNPs were added into the chitosan emulsion for 30 min before adding PVA to the mixture and then were mixed to ensure uniform dispersion before pouring into petri plates. Likewise for hydrogel incorporating GT-based AgNPs (GT-HG), GT-based AgNPs were added to the hydrogel mixture. Similarly, the AgNPs synthesized from *Aloe Vera* were added to the hydrogel mixture to obtain hydrogel incorporating *Aloe Vera*-based AgNPs (AV-HG).

### Characterization of AgNPs hydrogel wound healing material

#### Morphological characterization

Morphological examinations of the hydrogel films and microstructure observation of the porous AgNPs were performed by scanning electron microscope (SEM) (Te-scan, vega3). All the hydrogels were staged on a metallic stub adhered with double-sided tape and further coated with a golden layer.

### Energy dispersive X-ray (EDX) analysis

Energy dispersive X-ray spectroscopy (EDX) (Quanta FEG250) was used as a semi-quantitative analysis of AgNPs to confirm the presence of biosynthesized AgNPs in hydrogel patches based on the energy and intensity distribution of X-ray signals generated by the electron beam striking the surface of the specimen. Liquid nitrogen was used to cool the EDX chamber during the analysis.

### Mechanical properties

Preventing wound dressing displacement and pain during muscle activity, a wound dressing material’s tensile property should be like those of human skin. The hydrogel films were analyzed for mechanical properties (tensile strength (N/mm^2^) and elongation at break (%) by texture analyzer (Instron 34 sc^− 5^) with 5 kg of loaded cells. One cm^2^ film was cut and clutched between the clamps, then separated at a rate of 50 mm/min [1].

### Thickness and weight variations

The thickness of the hydrogel patches was measured using a digital calibrated micrometer. Three readings were taken, and then their averages and standard deviations were recorded. The weight variation testing included weighing each patch individually and the average weight ± standard deviation (SD) was calculated. All evaluations were performed in at least 3 replicates [1].

### Differential scanning calorimetry (DSC)

Differential scanning calorimetry (DSC) is a popular technique for characterizing hydrogels by presenting the melting temperature and enthalpy of fusion. It is a sensitive technique for measuring polymer transitions as a function of varying temperatures using heat capacity changes. Hydrogels were sealed in aluminum pans and heated at ambient temperature (50–300 °C) at a pre-programmed rate of 10 °C/min.

### Moisture content

The moisture content of the hydrogel films was determined using the following procedure. First, the initial weight (Wi) of each film was recorded. Then, the films were placed in a desiccator containing activated silica gel for 24 h. The films were weighed repeatedly at intervals until a constant weight (Wd) was reached [1]. The following equation Eq. (1) was used to calculate the moisture content (%):

1 $$\:Moisture\:Content\left(\%\right)=\frac{{W}_{i}-{W}_{d}}{{W}_{d}}\times\:100$$

### The porosity of hydrogel/ AgNPs patch

The porosity of the patches was measured using the alcohol displacement [15]. Patches (1 cm x 1 cm) were soaked in 1 mL of ethanol until saturated. After 24 h, the materials were removed and weighed. The formula for calculating porosity (P) is as follows Eq. (2):

2 $$\:\text{{\rm\:P}}=\frac{{W}_{2}-{W}_{1}}{\rho\:{\upsilon\:}_{1}}$$

Where W1 and W2 are the weights of the patches before and after immersion, 𝜐1 is the alcohol volume before immersion, and ρ is the constant density of alcohol [16].

### The swelling ratio of hydrogel/agnps patch

The hydrogel/AgNPs patch was cut into square-shaped specimens (1 cm x 1 cm). The specimens were weighed and immersed in 250 mL of phosphate buffer (pH 7.4) at 25 °C. At predetermined intervals, the patch specimens were weighed after being blotted with tissue paper to remove surface water. The swelling ratio was calculated using the following formula Eq. (3):

3 $$\:Swelling\:ratio\:\left(\%\right)=\frac{\left({W}_{s}-{W}_{d}\right)}{{W}_{d}}\times\:100$$

$\:{W}_{d}$ represents the initial weight of dry patch specimens, and $\:{W}_{s}$ represents the weight of swollen patch specimens. The experiment was carried out in triplicate [16].

### WVTR (Water vapor transmission Rate)

WVTR ensured that the hydrogel/AgNPs patch were kept in a moist environment over a wound. The patch was mounted on top of a polytop glass (144 mm^2^) that contained 10 mL of phosphate buffer (pH 7.4). The patch was weighed before being placed in an oven at 35 °C for 24 h. WVTR was calculated using the equation shown below Eq. (4):

4 $$\:WVTR\left(g/m2/day\right)=\frac{{W}_{i}-{W}_{t}}{A}\times\:{10}^{6}$$

Where A is the polytop opening area (mm^2^) and Wi and Wt are the polytop weights before and after the oven, respectively. The experiment was conducted in triplicate [1].

### Evaluation of hydrogel/agnps patch stability

The degradation kinetics under physiological conditions were assessed using the stability assay described below. 8.5 mL of Ringer’s solution, which contained 8.6 mg/mL of NaCl, 0.3 mg/mL of KCl, and 0.33 mg/mL of CaCl_2_ are added to the hydrogel/ AgNPs patch, incubated at 37 °C for 30 days. After incubation, the supernatant was removed. The weight loss percentage was calculated using the following formula Eq. (5):

5 $$\:\varDelta\:W\left(\%\right)=\frac{{W}_{0}-{W}_{t}}{{W}_{t}}\times\:100$$

Where ($\:{W}_{0}$) is the weight of the patches before adding Ringer’s solution and ($\:{W}_{t}$) is the weight of the patches after it was removed [17].

### Biodegradability test of hydrogel/agnps patch

The biodegradation of the hydrogel patches was assessed by measuring the weight of loss percentage after planting it in the soil and the weight it also before planting and after removing it from the soil, and the percentage of weight loss after 30 days was calculated by the following equation Eq. (6) [18].

6 $$\:{W}_{loss}\left(\%\right)=\frac{{W}_{1}-{W}_{2}}{{W}_{1}}\times\:100$$

### In vitro drug release

The hydrogel/ AgNPs patch was added to a beaker to take a specimen of it. The assembly was securely placed at the bottom of the dissolution test apparatus, using a pH 7.4 phosphate buffer as dissolution media at room temperature. A UV/Vis spectrophotometer was used to measure the concentration of AgNPs released from aliquot specimens that were collected at pre-arranged intervals. In vitro drug release data was collected in order to gain a better understanding of the mechanism of drug release from the formulation of wavelength and the absorbance of the AgNPs [1].

### Cytotoxicity test

A total of 10^4^ cells/well of HSF (human skin fibroblast) cell lines, purchased from Nawah scientific Inc., (Mokatam, Cairo, Egypt), were cultured and seeded in a 96-well plate for 24 h in Dulbecco’s Modified Eagle Medium (DMEM) containing 10% Fetal bovine serum (FBS) incubated at 37 C and 5% CO_2_. Then, the medium was changed with a DMEM with 10% FBS containing concentrations of hydrogel emulsion and incubated for 24 h 37 °C with 5% CO_2_. The cells in a free medium serve as a negative control. Next, the medium changed with 3-[4,5-dimethylthiazol-2-yl]-2,5 diphenyl tetrazolium bromide (MTT) (5 mg/mL) for 4 h. The readings were measured at 570 nm after dissolving the formazan using Dimethyl sulfoxide (DMSO) [8].

### In vitro antimicrobial assessment using agar diffusion test

Gram-negative *E. coli* and Gram-positive *S. aureus* were chosen as model microorganisms. Overnight cultures of E. coli and S. aureus were prepared by plating and transferring them to broth for growth at 37 °C. The cultures were then diluted and incubated in fresh broth to achieve a specific optical density of 0.5 McFarland Standard (about 1 × 10^8^ CFU/mL). These bacterial suspensions were uniformly spread onto LB agar plates. Hydrogel discs, ensuring consistent weight and shape, were applied to the agar surface. After incubation at 37 °C, the inhibition zones around the hydrogels were measured to assess their antimicrobial activity, Following overnight incubation of inoculated plates, the zones of inhibition corresponding to the lowest inhibitory concentrations were investigated [8]. The experiment was replicated three times for each bacterial strain. Hydrogels with different concentrations of AgNPs were used (0.5% for A1, 0.8% for A2, 0.5% for G1, 0.8% for G2, 0.5% for C), and the control hydrogel (Cr-HG) was also used.

### Statistical analysis

ANOVA was used to perform statistical analysis using SPSS Statistics. The mean and standard deviation of all data were determined. Statistical differences with a p-value ≤ 0.05 were considered significant.

## Results and discussion

### Characteristics of AgNPs

#### UV-Vis spectrophotometer

**Fig. 2:**
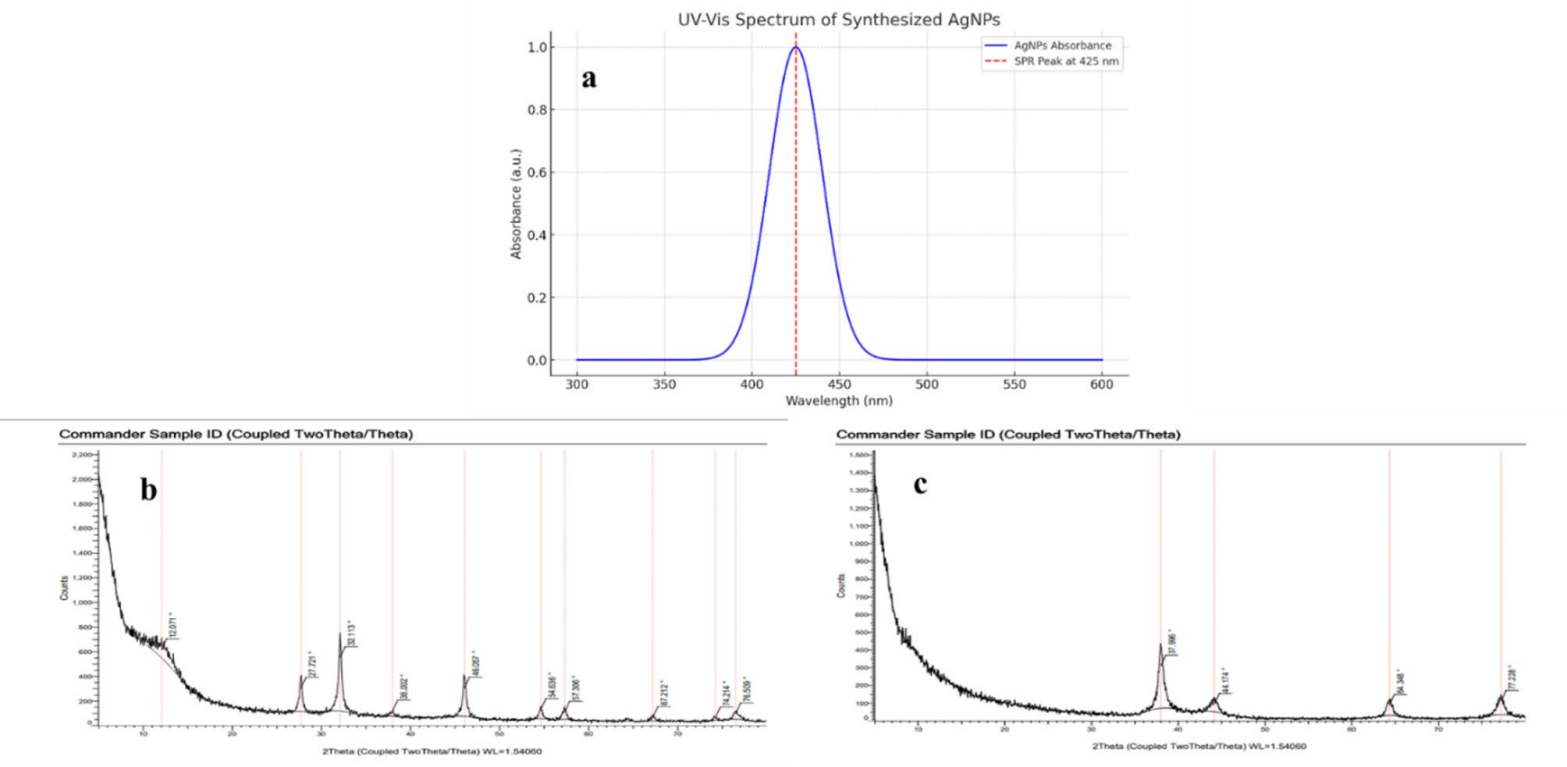
**(a)** UV-VIS absorption spectra of synthesized AgNPs. The X-ray diffraction (XRD) patterns for AgNPs for **(b)** (AV-HG), and **(c)** (GT-HG)

UV-VIS spectrophotometer was carried out to ensure the formation of the synthesized AgNPs, as reported previously [19]. Wilson’s earlier report on the efficient synthesis of green-synthesized AgNPs was validated by their optical response, which was attributed to their intense surface plasmon resonance band in the spectral range [20]. A color change has been observed in the mixture of plant extract and AgNPs. The absorbance of the solution was investigated. From the spectral analysis, the AgNPs peak was at 425 nm, Fig. 2(a) indicating the presence of spherical AgNPs. This supported fairly similar results from other previous studies, where the existence of the SPR band maxima in the corresponding UV-Vis absorption spectra in the region of 410–460 nm was linked to the production of spherical and/or near-spherical AgNPs [21].

#### XRD

XRD analysis for AgNPs by *Aloe Vera* (AV-HG) determined the crystallographic structure of the green synthesized AgNPs, Fig. 2b. The XRD pattern displayed different peaks identified at 2θ were (37.996°), (44.174°), (64.348°), and (77.228°), corresponding to the (111), (200), (220), and (311) planes, respectively. The face-centered cubic (fcc) structure of the biosynthesized AgNPs, with the 38° peak being the most intense, Fig. 2. This indicates high purity and good crystalline quality and suggests the predominant crystalline orientation around the (111) plane. The sharpness of the peaks implies a uniform particle size distribution. For AgNPs by green tea, the XRD graph has more peaks compared to the AgNPs by *Aloe Vera* graph, Fig. 2(c). This could be due to the specimen having multiple crystallographic phases, each contributing additional peaks. The most prominent peaks are likely to correspond to the planes of atoms in the crystal structure, the peaks are (32.113°), (46.057°), (67.212°) and (76.509°). This result indicates that AgNPs by *Aloe Vera* (AV-HG) graph peaks align with the crystalline nature of the AgNPs [22].

### Characterization of hydrogel patches

#### Scanning electron microscope (SEM) imaging of hydrogel patches

**Fig. 3:**
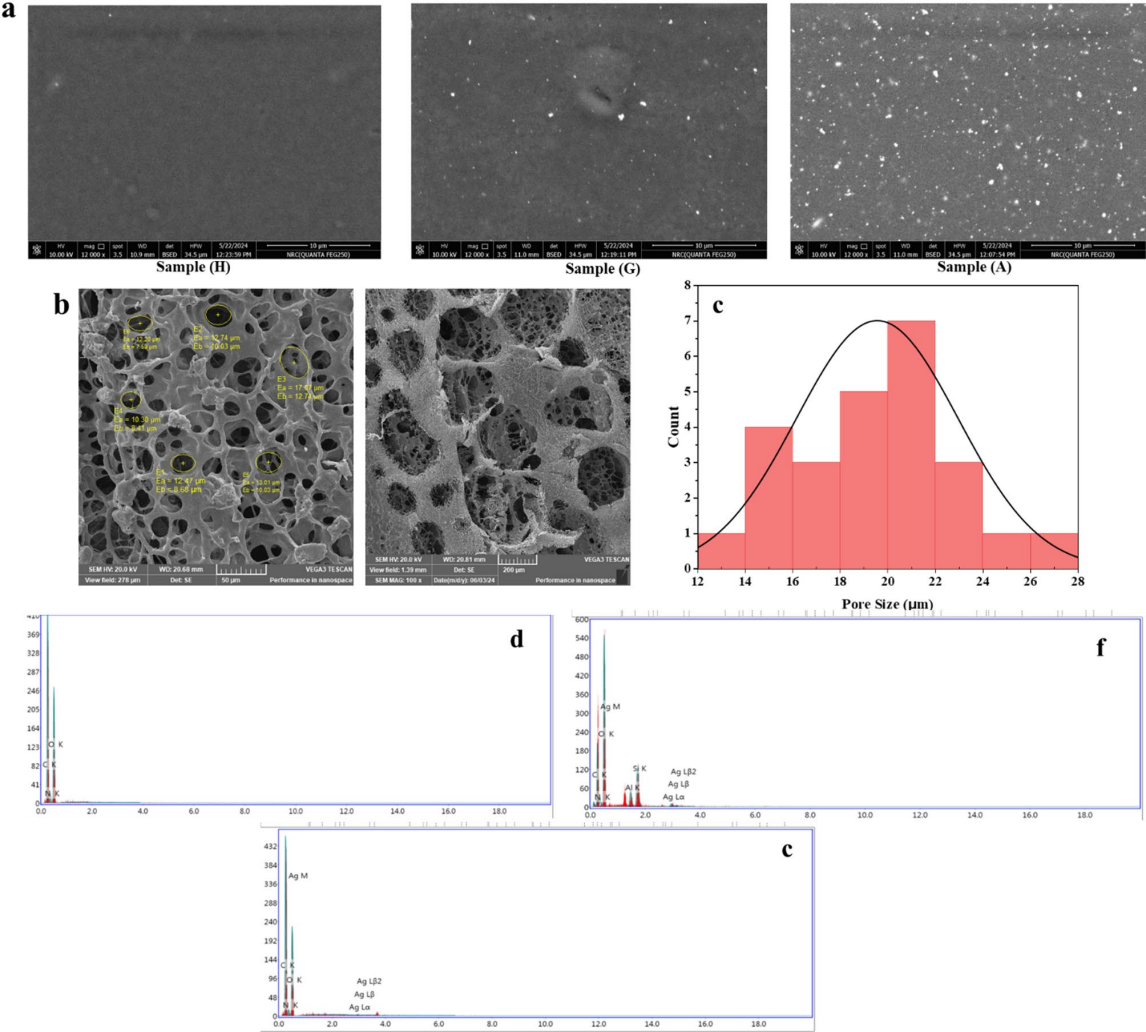
**(a)** SEM images for the surface of the hydrogels: (Cr-HG), (GT-HG), and (AV-HG). **(b)** SEM for hydrogel film (AV-HG) after freeze drying and (c) its histogram profile. EDX spectrum for the three hydrogels **(d)** (Cr-HG), **(e)** (AV-HG), and **(f)** (GT-HG)

SEM images demonstrated the surface morphology of the hydrogel patches for (Cr-HG), (AV-HG), and (GT-HG) as shown in Fig. 3(a). For (AV-HG) and (GT-HG), the bright spots corresponding to the AgNPs indicate their successful incorporation into the hydrogel matrix. The well-dispersed and relatively small nanoparticles in the (AV-HG) indicate an effective synthesis and stabilization process, resulting in a uniform distribution without significant aggregation. In contrast, (GT-HG) showed aggregation of AgNPs. These results indicate that (AV-HG) has a smoother surface and is more uniformly distributed than (GT-HG). (Cr-HG), exhibited a homogenous matrix with a smooth surface. Figure 3(b) shows hydrogel after freeze drying the range of size for AgNPs and the morphology of the composition of (AV-HG), and these results align with another previous study [1].

The porous behavior of hydrogel patch is a critical characteristic influencing its wound healing properties. SEM images, Fig. 3(b), reveal the interconnected network of micropores with irregular shapes. As well the quantitative analysis of the SEM images, based on the measurement of average pore size, demonstrated a heterogeneous pore size distribution the equivalent diameter of the pores ranged from approximately 6 μm to 27 μm as observed from histogram profile (Fig. 3(c)). This porous structure of hydrogel has a significant impact on its wound healing properties as it creates an environment to accelerate wound healing process [23].

### EDX

The elemental constituents and relative abundance of the green synthesized AgNPs loaded on a hydrogel patches (AV-HG) and (GT-HG) were determined using Energy Dispersive X-ray (EDX) as presented in Fig. 3(c-e). The EDX spectrum Fig. reveals the purity and the complete elemental composition of AgNPs. The elemental constituents and relative abundance of the control hydrogel (Cr-HG) were also analyzed Fig. 3(c). Figure 3(d) reveals the EDX analysis for (AV-HG) showed the percentage relative composition of elements such as oxygen (O) 59%, silicon (Si) 4%, aluminum (Al) 2%, nitrogen (N) 5%, carbon (C) 26%, and silver (Ag) 4%. The percentage of silver evidenced by the detectable silver peaks confirms that the nanoparticles were synthesized successfully. The results indicate that nanoparticles may be dispersed throughout the organic matrix provided by the *Aloe Vera*. Figure 3(e) reveals the EDX analysis for (GT-HG) showed the percentage relative composition of elements such as oxygen (O) 44%, nitrogen (N) 9%, carbon (C) 45%, and silver (Ag) 2%. Our findings suggest that (AV-HG) exhibit greater potential for high-yield AgNPs synthesis. In contrast, (GT-HG) does not show significant impurities. Overall, (AV-HG) might be preferable to its increased yield of AgNPs.

The observed high yield of silver nanoparticles using *Aloe Vera* extract compared to green tea can be attributed to several reasons as follows. Firstly, the difference in the composition of *Aloe Vera* and green tea extract which affects their activity as reducing agents. While both *Aloe Vera* and green tea contain polyphenols, *Aloe Vera* is also rich in polysaccharides, vitamins, and amino acids, which may contribute to a more efficient reduction of silver ions [24]. Furthermore, the high concentration of acemannan (natural polysaccharide) in *Aloe Vera* may lead to faster reduction of kinetics and improved stabilization of the formed AgNPs [25]. The UV-Vis spectroscopy data for the *Aloe Vera* reaction, suggesting a higher concentration of AgNPs compared to studies using green tea.

### Mechanical properties of hydrogel patches

**Fig. 4:**
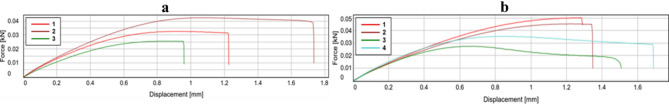
Mechanical properties graph for hydrogels **(a)** AV-HG and **(b)** Cr-HG, (*n* = 3)

The mechanical properties of the hydrogel were evaluated to determine whether it is appropriate for practical application in wound healing. To determine the tensile strength of the hydrogels uniaxial tensile tests were performed on a 1 cm^2^ specimen. After entering the texture analyzer clamps, the specimens were continuously pushed apart at a speed of 50 mm per minute. (AV-HG)’s tensile stress at a tensile strength of (67.13) MPa demonstrates the hydrogel’s excellent mechanical integrity and capacity to withstand large strains without breaking. Elongation at break and tensile strength were measured. This property shows the ductility and flexibility of hydrogel. High ductility and flexibility were indicated by the hydrogel displacement from tensile strain (elongation) at a break of 4.34%. This suggests that before breaking it can undergo significant deformation. Furthermore, (69. 14) MPa of tensile strength is maintained by the hydrogel according to (Cr-HG) results. This shows the hydrogel has a high mechanical strength and the ability to withstand stress before breaking. Given that the hydrogel has a tensile strain displacement (elongation) upon break of 4.84%, one crucial property that suggests it may undergo significant deformation before breaking is its high tensile strength. These results imply that both hydrogels mechanical qualities are promising however (AV-HG) in Fig. 4(a); Table 1 breaks more slowly and has a greater displacement than (Cr-HG) in Fig. 4(b); Table 1. A good mechanical quality was demonstrated by chitosan membranes mixed with PVA for use in medical products and the regulated release of nanoparticles.

**Table 1:** The mechanical properties of hydrogels (AV-HG and Cr-HG)

Parameter	Mean (AV-HG)	Standard Error (SE) (AV-HG)	Mean (Cr-HG)	Standard Error (SE) (Cr-HG)
Force at Break (Standard) [kN]	0.03	0.00057	0.03	0.01
Tensile stress at Break (Standard) [MPa]	63.89	7.62	69.14	16.255
Tensile strain (Displacement) at Break (Standard) [%]	4.34	0.75	4.84	0.295
Time at Break (Standard) [s]	31.91	10.8	15.64	2.745
Tensile displacement at Break (Standard) [mm]	1.30	0.225	1.45	0.09
Modulus (Automatic Young’s) [MPa]	4226.92	243.72	4768.05	248.095
Force at Tensile strength [kN]	0.03	0.00057	0.04	0.0005
Tensile stress at Tensile strength [MPa]	67.13	9.74	79.02	10.195
Tensile strain (Displacement) at Tensile strength [%]	3.14	0.207	3.36	0.5
Tensile displacement at Tensile strength [mm]	0.94	0.063	1.01	0.15

### Thickness and weight variation

The thickness of the hydrogel patch was measured multiple times using a calibrated micrometer. Three measurements from various locations within each patch were taken to ensure accuracy. The average thickness of 0.0436 ± 0.0045 mm for the AgNPs hydrogels (AV-HG) showed a highly homogeneous patch development with minimal fluctuations. The average thickness of the control (Cr-HG) was 0.0473 ± 0.0082 mm showing uniform thickness throughout the patches. A weight variation analysis was conducted for each hydrogel patch. The AgNPs hydrogels weight was found to be extremely uniform, averaging 0.06587 ± 0.0108 mm. 0.6226 ± 0.1994 mm was the average weight of the control which showed better weight consistency than the AgNPs hydrogels. These values are not too different from the previous study of CS-PVA hydrogel films which could mean that the addition of AgNPs to the CS-PVA hydrogel did not affect these characteristics [1].

#### Differential scanning calorimetry (DSC) analysis

**Fig. 5:**
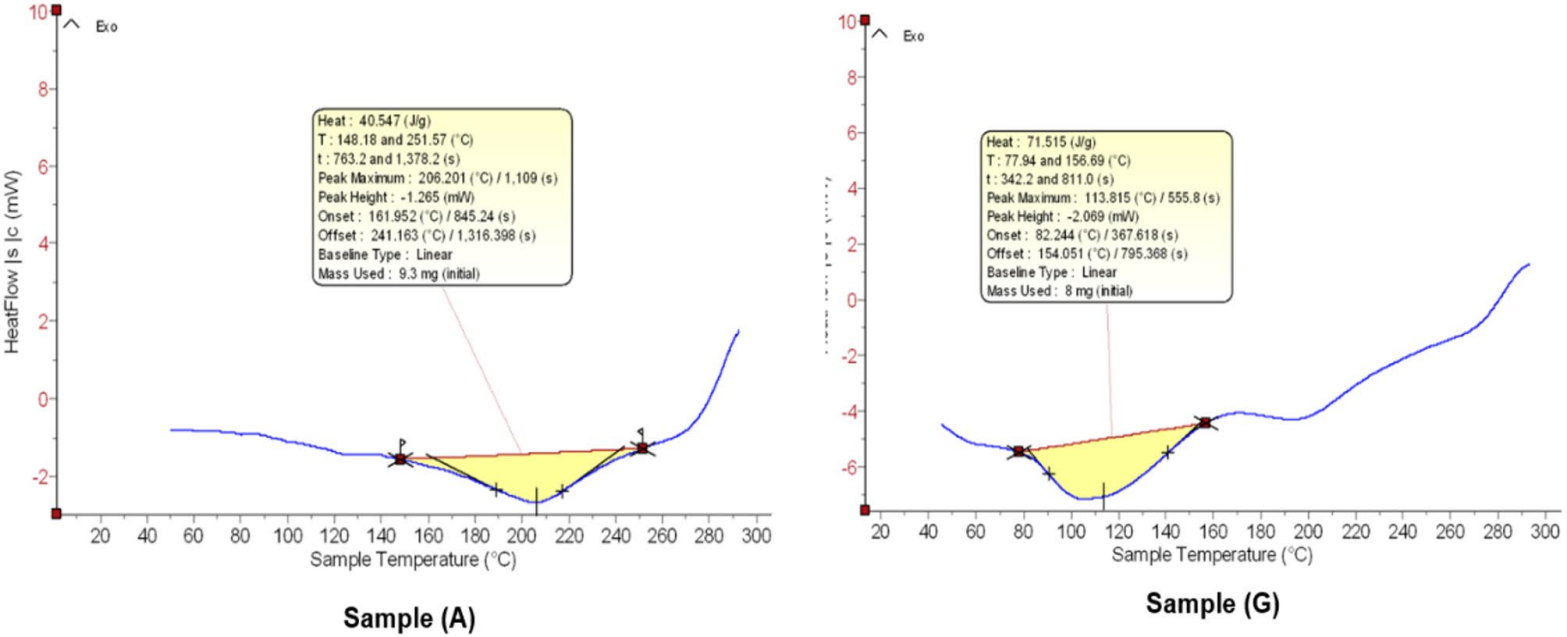
DSC graphs of (A) for (AV-HG) and (G) for (GT-HG) that show hydrogel temperature (X axis) and Heat flow (Y axis)

The DSC was employed in our investigation to comprehend our hydrogel patches heat conduct and the stability with thermal increase. The DSC curve for hydrogel (GT-HG), Fig. 5, shows an endothermic peak with an enthalpy (∆H) of 71.52 J/g, a maximum at 113.82 °C, an onset at 82.24 °C, and an offset at 154.05 °C due to loss of adsorbed water in the hydrogel due to its hydrophilic nature. This alteration suggests that crystalline areas of melting and the hydrogel matrix dehydrates, highlighting the resilience of the composite in this temperature range. On the other hand, the hydrogel (AV-HG) has a reduced enthalpy (∆H) of 40.547 J/g and a large endothermic peak at 208.00 °C. Its onset and offset temperatures are 161.62 °C and 241.76 °C, respectively. These results indicate a significant thermal event, likely the melting or the decomposition of organic components from *Aloe Vera*, within a wider temperature range, with the hydrogel (AV-HG) showing higher thermal stability. These variations in thermal properties between (AV-HG) and (GT-HG) suggest differences in their structural integrity and energy absorption processes, influenced by different organic components of *Aloe Vera* and green tea.

#### Moisture content & WVTR

For optimal and ideal wound healing, maintaining a moist environment is crucial, necessitating a careful balance between WVTR and moisture content. As it was found that one of the main issues for patients suffering from serious skin defects is total wound surface dehydration [15]. In the present study, the control showed a higher WVTR of 2777.78 g/m² day and a moisture content of 10.45%. Indicating their suitability to be used for transporting extrudes from the surrounding skin and maintaining ventilation. It can also help to reduce the risk of maceration and infection of wound and tissue [26]. In contrast, the hydrogels integrated with AgNPs showed a lower WVTR of 1388.89 g/m² day and a moisture content of 7.73%. However, it has been suggested that the optimal WVTR for a wound dressing should be between 2000 and 2500 g/m² per day to avoid both excessive drying of the wound and the accumulation of exudates, which can promote bacterial growth. Consequently, while the incorporation of AgNPs may improve other characteristics of the hydrogel, the low WVTR could reduce its effectiveness as a wound dressing by increasing the risk of exudate accumulation and infection [15]. As can be seen, a slight decrease in WVTR occurs when AgNPs are added to the CS/PVA hydrogel. The longer diffusive path that molecules must take through the nanocomposite hydrogel in the presence of the fillers (as AgNPs) which are referred to as tortuosity, is the cause of the decrease in the diffusion rate of water vapor [27]. This characteristic has been demonstrated in earlier research on hydrogels using nano-fillers [28]. Pelissari et al. discovered that in hydrogels containing cassava starch and CS, WVTR decreased as CS concentration increased because hydrogen bonds formed between the NH_2_ of CS and cassava starch’s OH, decreasing the hydrophilic groups’ availability [29]. Similar results were reported by Feiz and Navarchian in the same pattern in a PVA/Chitosan-modified Clay nanocomposite hydrogel, where the addition of 5 weight% filler caused the WVTR to drop to roughly 37.5% [30].

### The porosity of hydrogel/ AgNps patch

Porosity was determined using the alcohol displacement method [15]. The control hydrogel had a porosity of (0.67%) while the integrated hydrogel had a higher porosity of (1.08%). Since exchange and fluid absorption are essential for efficient wound healing, the AgNPs patches increased porosity suggests improved fluid absorption and gas exchange capabilities and causing the molecules to transport through it fast enough. According to these findings the AgNPs patches have the potential to enhance patient comfort because of their greater flexibility to maintain a more ideal moist environment and enhance oxygen transport to the wound site [31]. The the high porosity structure of the AgNPs patches and the good bactericidal action of AgNPs work together to provide integrated hydrogel’s outstanding wound healing effect [32, 33]. Similarly, a study on alginate control bandages and nZnO-incorporated composite bandages observed similar porosity changes with nanoparticle addition. This indicates that while the absolute porosity values differ due to the inherent differences in material composition. The observed increase in porosity with the incorporation of nanoparticles is a consistent finding across different studies [16].

### The swelling ratio of hydrogel/agnps patch

**Fig. 6:**
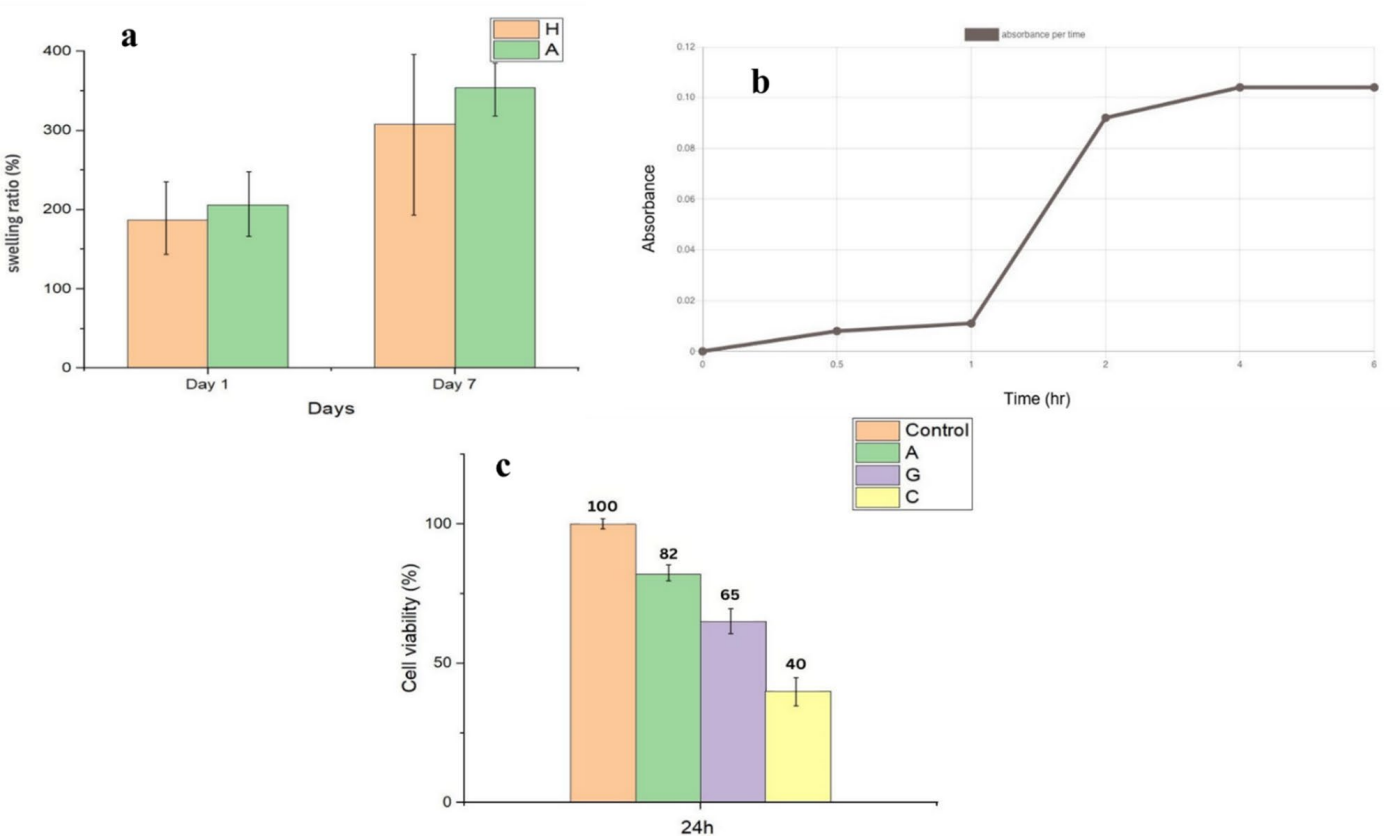
**(a)** Swelling ratio of blanket (Cr-HG) and (AV-HG) contained AgNps patches (*n* = 3). **(b)** UV-Visible Spectra of Optimized the absorbance of the AgNPs in the patches (X-axis: Time (hour), Y-axis: Absorbance). **(c)** Cell viability of control (Cr-HG), A = *Aloe Vera* patch AgNPs (AV-HG), G = green tea patch AgNPs (GT-HG), C = chemical patch AgNPs (Ch-HG) after 24 h

A key characteristic for understanding the wound healing property of hydrogel films is the fluid uptake capacity, which was studied through evaluation of the swelling ratio. Hydrogel wound patches should have large water absorption capacity in order to absorb wound exudates [33]. The initial swelling ratio that was measured after one day, Fig. 6(a), for the control (Cr-HG) was 186.61%, while the hydrogels containing green-synthesized AgNPs (AV-HG) showed a higher initial swelling ratio of 206.02%. After one week, the swelling ratio of both types of patches increased, with the control hydrogel reaching 307.9% and the AgNPs hydrogel reaching 354.21%. The higher swelling ratios indicate that AgNPs hydrogel patches can maintain a moist environment more effectively than the control hydrogel patches, which is beneficial for promoting wound healing. These findings align with previous studies that also highlight the importance of the swelling ratio in the context of hydrogel applications [34].

### Evaluation of hydrogel/agnps patch stability

The long-term stability of hydrogel films for hydrogels (Cr-HG) and (AV-HG) in physiological conditions, which is critical for biological applications, was assessed. For (AV-HG), the weight loss% after 30 days was approximately 71.22% and this result indicates that the degradation level of the *Aloe Vera*-synthesized AgNps provides structural stability to the hydrogel matrix and maintains its integrity for an extended period. In contrast, the (Cr-HG) degraded completely within the same 30-day period, suggesting it lacks the necessary structural stability under physiological conditions. These results highlight the important role of AgNPs in enhancing the durability and stability of the hydrogel matrix, which reduces the degradation rate of the nanocomposite hydrogel [35, 36]. In general, the hydrogel’s degradation rate is directly proportional to its swelling ratio; that is, the lower the swelling ratio, the slower the hydrogel will degrade [35, 37] which confirms the previously obtained data.

#### Biodegradability test of hydrogel/agnps patch

Biodegradation of the hydrogel patches was evaluated by measuring the percentages of the loss of weight over 30 days, (AV-HG) exhibited a (22%) loss in weight, compared to (Cr-HG) which lost 16% of its weight. These results suggest that the incorporation of AgNPs in (AV-HG) may accelerate the degradation process, with its additional benefits in the wound healing applications. These findings align with previous studies that were investigating the biodegradation of hydrogels with time [37].

### In vitro drug release

To measure the release of the AgNPs, we measured the absorbance from a UV spectrophotometry at 420 nm. After half an hour, the absorbance was weak (0.008). But after 1 h, it was increased to (0.011). Absorbance was measured again after 2 h and increased to (0.029) and after 4 h it was (0.104). This number was also fixed after 5 h so the release became fixed and increased over time as shown in Fig. 6(b). In another study, the peak of AgNPs was observed at 478 nm [11], while in our study, it stabilized at 420 nm. For wound healing, smaller and more uniformly dispersed AgNPs are preferred due to their higher surface area and enhanced antimicrobial properties. Therefore, a peak at 420 nm would be better suited for wound healing applications. These nanoparticles are likely to be more effective in preventing infections and promoting healing due to their increased surface interaction with microbial cells and biological tissues.

#### Cytotoxicity test

Biocompatibility and low cytotoxicity are imperative requirements for wound healing dressing. AgNPs are known to be potentially toxic to bacteria and cells. The vitality of dermal fibroblasts in direct contact with hydrogels (AV-HG) that with AgNPs was investigated to determine by MTT test. The results showed a slight decrease in cell vitality, 82% after 24 h, for those that contain AgNPs of *Aloe Vera* and that place it as non-toxic, Fig. 6(c). However, as was expected, the cell viability decreased in the presence of AgNPs from the green tea. The cell viability for (GT-HG) was 65%, which places hydrogel at the threshold of low cytotoxicity. The hydrogel with chemical AgNPs (Ch-HG) showed a cell viability of around 40% determined as highly toxic due to the lower cell viability, all of them calculated due to the absorbance of the control (Cr-HG) that 100% cell viability. A similar result was found in [8], our results show lower toxicity than any other paper due to the extract method leading to higher efficacy with lower concentrations so as shown in antimicrobial activity the most powerful conc is the 0.5%.

### In vitro antimicrobial assessment using agar diffusion test

**Fig. 7:**
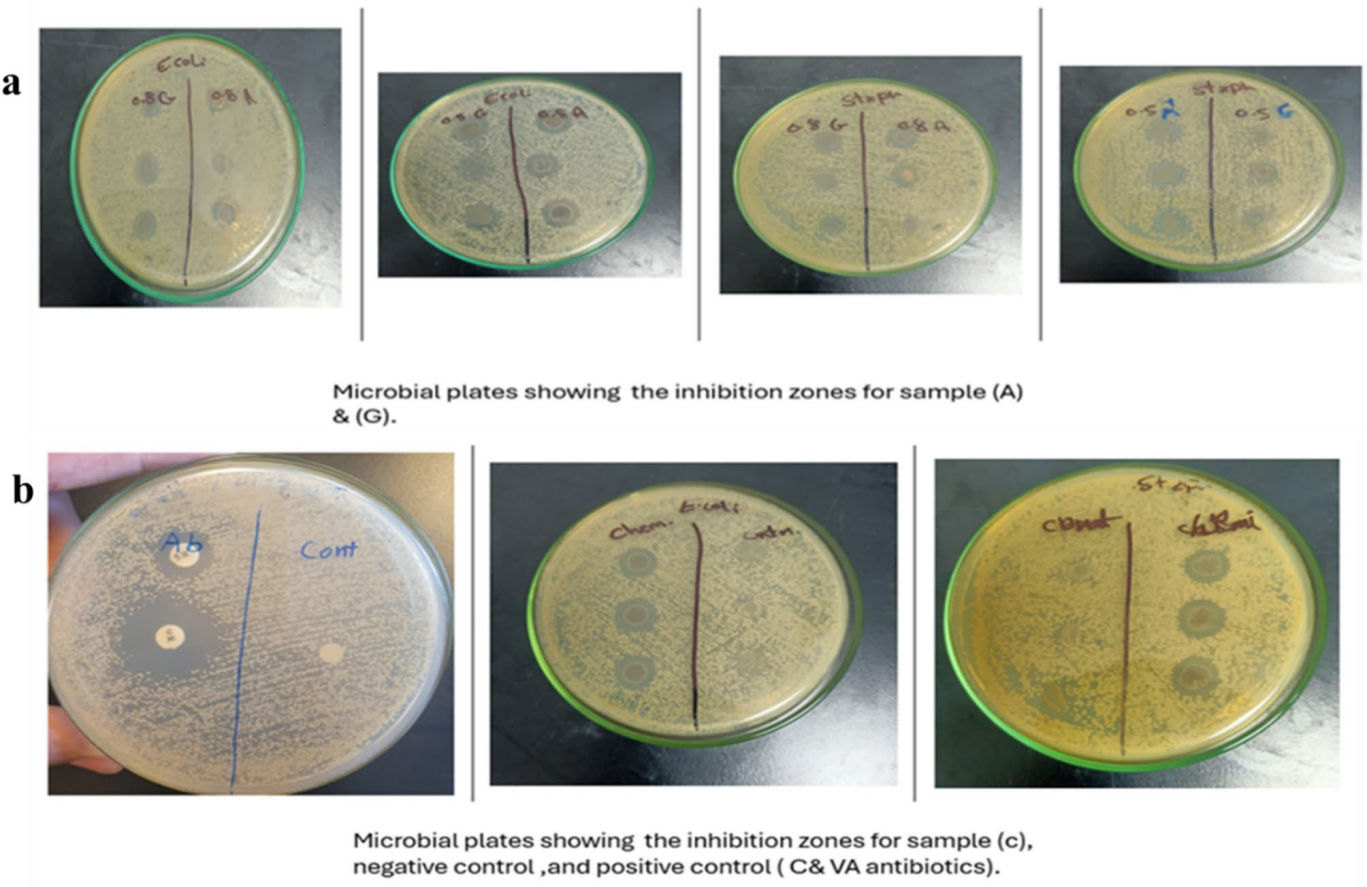
**(a)** Anti-microbial activity of AV-HG and GT-HG with concentration 0.5% and 0.8% of the patches. **(b)** Anti-microbial activity of Ch-HG and Cr-HG (control)

The antibacterial activity of hydrogel films was investigated against *Staphylococcus aureus* and *Escherichia coli.* The zone of inhibition for different hydrogel is represented in Fig. 7(a). The negative control hydrogel (Cr-HG) which did not contain any AgNPs, showed no inhibition zones. For *S. aureus*, the positive control using vancomycin (VA) and chloramphenicol (C) antibiotics showed 0.6 cm and 1.2 cm zones of inhibition respectively, and for *E. coli* the positive control using (VA) and (C) antibiotics showed 0.8 cm and 1 cm zones of inhibition, respectively. This indicates the effectiveness of antibiotics against bacterial strains. The chemically synthesized AgNPs (Ch-HG) presented moderate inhibition zones against both bacteria: 0.4332 cm with *S. aureus* and 0.575 cm with *E. coli*, indicating their efficacy in inhibiting bacterial growth. However, the green synthesized AgNPs (GT-HG), particularly those synthesized using *Aloe Vera* (AV-HG), presented larger inhibition zones, and thus superior antibacterial properties. For *S. aureus*, (AV-HG) at a concentration of 0.5% presented an inhibition zone of 0.675 cm and 0.65 cm at a concentration of 0.8%. For *E. coli*, the inhibition zones were 0.666 cm and 0.7 cm for the 0.5% and 0.8% concentrations, respectively. This result indicates that the presence of bioactive compounds in *Aloe Vera* contributes to the improved stability of the AgNPs, enhancing their antimicrobial properties. (GT-HG) showed lower inhibition zones compared to (AV-HG) at the same concentrations. For *S. aureus* the inhibition zones were 0.425 cm and 0.45 cm for the 0.5%, and 0.8% concentrations, respectively. For the *E. coli*, the inhibition zones were consistent at 0.475 cm at both concentrations, Fig. 7(b). This suggests that the antibacterial effect of green tea-synthesized AgNPs (GT-HG) does not significantly increase with increasing concentration of AgNPs.

The antibacterial mechanism of action can be divided into sever*a*l steps. Firstly, the release of entrapped AgNPs from the PVA/chitosan (PVA/CS) hydrogel networks, which includes diffusion of the nanoparticles through the hydrogel matrix, swelling of the hydrogel matrix facilitating release, and controlled release of AgNPs. Secondly, the released AgNPs will attract sulfur-containing proteins in the bacterial cell wall and cytoplasmic membrane. Due to their nanoscale size, they will penetrate bacterial cell walls [38]. After the uptake of free silver nanoparticles into cells, respiratory enzymes can be deactivated, leading to the generation of reactive oxygen species (ROS). These ROS can disrupt the cell membrane and induce DNA modifications [39]. Moreover, silver ions can inhibit the synthesis of proteins by denaturing ribosomes in the cytoplasm. The denaturation of cytoplasmic membrane can rupture organelles and even result in cell lysis [40]. In addition, AgNPs can be involved in bacterial signal transduction. Bacterial signal transduction is affected by phosphorylation of protein substrates, and nanoparticles can dephosphorylate tyrosine residues on the peptide substrates. Disruption of the signal transduction can lead to cell apoptosis and termination of cell multiplication [41].

According to subsequent studies maintained on *Aloe Vera* extract for identifying and quantifying its phytocomponents using gas chromatography–mass spectrometry GC–MS maintained on Aloe *Vera* gel and its extract [42]. It was shown that extract contained volatile compounds such anthraquinone glycosides, phenolic compounds and esters. These components are noted for their antimicrobial activity. The extract form included Tridecanoic acid (CAS) Decanoic acid, Methyl ester, 2.2-Dimethyl-1-pyrazylpropanol and eicosyl acetate, which specifically exhibit antibacterial activity. Such compounds have been linked to inhibiting the growth of various pathogens. Where the overall profile of green tea shows fewer volatile compounds as alkaloids, phenolics, flavonoids, saponins and steroids that are specifically linked to antimicrobial activity but with a fewer quantity than those present in *Aloe Vera*. *Aloe Vera*’s potent antimicrobial constituents are notably linked to its effectiveness against a wider array of pathogens, thereby enhancing its overall microbial activity. Therefore, the comparative low microbial activity of green tea can be attributed to the presence of fewer or less impactful antimicrobial phytochemical compounds when examined alongside the stronger and varied antimicrobial effects of the volatile compounds found in *Aloe Vera* [43].

Our results align with previous studies, demonstrating that the incorporation of bioactive compounds significantly enhances antibacterial efficacy against *S. aureus* and *E. coli* [3]. This highlights the potential of green synthesis methods for developing effective antimicrobial wound dressings.

Comparative studies of green AgNPs of current and previous works have been reported in Table 2 to clarify the key novelty of our work. Moreover, Comparative evaluation of green-synthesized AgNPs hydrogels for wound healing applications covering sizes, cytotoxicity, biodegradability/stability, moisture content has been reported in Table 3.

**Table 2:** Comparative analysis of antimicrobial activity and cytotoxicity of green-synthesized silver nanoparticles (AgNPs) from different plant extracts

Species used	Antimicrobial ZOI (mm)	Cytotoxicity	Ref
Green Tea Extract	8–11 mm (*S. aureus*, *Klebsiella*)	Not reported	[44]
Aloe Vera Extract	~ 20 mm (*E. coli*) at 0.3 M AgNO₃	No significant cytotoxicity on PBMCs	[6]
Neem Extract	17 mm (*P. aeruginosa*)	Not reported	[45]
Ginger Extract	MIC only: 0.187 mM (*S. aureus*), 0.375 mM (*E. coli*); no ZOI reported	Not reported	[46]
AV-HG, GT-HG	6.5-7 (AV-HG), 4.5–4.7 (GT-HG) Investigated S. aureus and E. coli	> 80% (AV), 65–75% (GT)	This Work

**Table 3:** Comparative evaluation of green-synthesized silver nanoparticles (AgNPs) hydrogels for wound healing applications

Hydrogel	NP Size (nm)	Antimicrobial ZOI (mm)	Cytotoxicity	Hydrogel Stability	Moisture Content	Ref
AgNPs loaded PVA/gum acacia HG	10–40	Not specified (tested on E. coli)	Not reported	Enhanced thermal stability	Swelling behavior influenced by gum acacia; good retention	[47]
CS HGs reinforced by AgNPs	10–20	19.97 ± 0.73 (S. aureus), 17.30 ± 0.03 (E. coli)	> 90% cell viability (HDF cells)	Ultrahigh mechanical stability (15.95 ± 1.95 MPa)	Swelling ratio ~ 41%	[48]
AgNPs impregnated CS-PEG HG	10–50	16 (S. aureus), 14 (E. coli)	> 85% cell viability	Mechanical strength (12.5 MPa)	Swelling ratio ~ 35%	[49]
CNF/G/Ag_0.5_ HG	~ 20	> 1 mm (S. aureus, P. aeruginosa)	≈ 100% infected cell viability (NHDF)	Self-recovery after 80% strain, stable over 30 days	WVTR = 2093.9 g/m²/day; high fluid and blood absorption	[50]
AV-HG, GT-HG	9–12 (AV), 12–15 (GT)	6.5-7 (AV-HG), 4.5–4.7 (GT-HG)	> 80% (AV), 65–75% (GT)	Stable in Ringer’s solution (72 h)	High retention (> 85% water content)	This work

## Conclusion

The silver nanoparticles were successfully synthesized by a rapid and environmentally friendly method, detecting the forming of AgNPs using a UV-spectrophotometer, Also with XRD their combination with the hydrogel and the nanoparticles size range and shape of particles was determined using SEM and the combination of element was determined by EDX. They also showed a spherical shape within nanoparticles better with the *Aloe Vera*. The films showed significant swelling, moisture content, porosity and mechanical properties which led to an ideal required for a good wound dressing formulation. They showed higher antimicrobial activity when synthesized from *Aloe Vera* than from green tea and were more stable in all tests with a quick release of silver that was measured by the in vitro drug release. The AgNPs *Aloe Vera* patches showed non-cytotoxicity and green tea showed low cytotoxicity. This study may be further extended to try to produce higher quality and better ways with more types of plants in the future and test it in vivo.

Furthermore, the study demonstrated that AgNPs *Aloe Vera* hydrogel patches achieved ideal swelling and mechanical properties with effective moisture retention as well as controlled porosity. Additionally, high antibacterial activity of AV-HG was showed against E. coli and S. aureus compared to GT-HG along with non-cytotoxicity toward human cells, highlighting their suitability for wound healing applications. These findings not only offer an eco-friendly solution for advanced drug delivery through wound dressings but also support the future growth of sustainable biomedical materials that can enhances healing outcomes and reduce healthcare costs.

## Data Availability

The datasets used and/or analysed during the current study are available from the corresponding author on reasonable request.

## Abbreviations

AgNO₃: Silver Nitrate
AgNPs: Silver Nanoparticles
AV: HG–Aloe Vera Hydrogel
CaCl₂: Calcium Chloride
Ch: HG–Chemically Synthesized AgNPs Hydrogel
Cr: HG–Control Hydrogel
CS: Chitosan
DMEM: Dulbecco’s Modified Eagle Medium
DMSO: Dimethyl Sulfoxide
DSC: Differential Scanning Calorimetry
EDX: Energy Dispersive X–ray Spectroscopy
fcc: Face–Centered Cubic
FBS: Fetal Bovine Serum
GT: Green Tea
GT: HG–Green Tea Hydrogel
KCl: Potassium Chloride
MTT: 3–[4,5–dimethylthiazol–2–yl]–2,5–diphenyl tetrazolium bromide
NaCl: Sodium Chloride
NaOH: Sodium Hydroxide
PVA: Polyvinyl Alcohol
SEM: Scanning Electron Microscopy
SPR: Surface Plasmon Resonance
UV: Vis–Ultraviolet–Visible Spectroscopy
VA: Vancomycin
WVTR: Water Vapor Transmission Rate
XRD: X–ray Diffraction
